# Screening for *Hepatozoon* parasites in gerbils and potential predators in South Africa

**DOI:** 10.4102/jsava.v88i0.1339

**Published:** 2017-02-08

**Authors:** D. James Harris, Ana Pereira, Ali Halajian, Wilmien J. Luus-Powell, Katlego D. Kunutu

**Affiliations:** 1 Centro de Investigação em Biodiversidade e Recursos Genéticos, Universidade do Porto, Portugal; 2 Department of Biodiversity, University of Limpopo, South Africa

## Abstract

Samples of gerbils and their potential predators were screened for the presence of *Hepatozoon* parasites (Apicomplexa: Adeleorina) using both microscopic examination and sequencing of partial 18S rRNA sequences. Positive samples were compared to published sequences in a phylogenetic framework. The results indicate that genets can be infected with *Hepatozoon felis*. A Cape fox was infected with *Hepatozoon canis*, whereas the sequence from an infected rodent fell within a group of parasites primarily recovered from other rodents and snakes.

## Introduction

Despite their obviously important role in ecosystems, information about the diversity and distribution of many parasite groups is still scarce. Molecular screening for parasites has the potential to greatly improve this knowledge, but has lagged behind advances in assessing free-living organisms (Giraud et al. 2008). However, this is changing as molecular markers become available to directly detect and identify parasites from host tissue samples.

Hepatozoonosis is a vector-borne infectious disease caused by intracellular haemogregarine parasites (Apicomplexa: Adeleorina), which has been particularly studied in domestic dogs and cats owing to their veterinary importance (Baneth 2011). The complex life cycle, involving invertebrate definitive hosts and one or more vertebrate hosts, has hindered studies of this group of parasites. Microscopic examination of gamonts in vertebrate blood cells led to the description of various species, but the use of molecular tools indicated additional complexities. For example, the traditional identification of *Hepatozoon canis* or *Hepatozoon americanum* in canids and *Hepatozoon felis* in felids is partially contradicted, with lions occasionally infected with parasites identified from analysis of rRNA gene sequences as *H. canis*, and hyenas infected with *H. felis* (Williams et al. 2014). This is important, as infection with some species of *Hepatozoon* may be relatively more detrimental to the health of the host (Baneth 2011). Therefore, knowing which *Hepatozoon* species are present has relevance for conservation of wild hosts and has veterinary implications in domestic animals. Additionally, molecular screenings have indicated that trophic pathways may explain the parasites found in both predators and prey, including rodent–snake (Tomé et al. 2014) and rodent–canid (Maia et al. 2014) food chains. At the same time, prevalence varies hugely between hosts and between study areas – for example Maia et al. (2014) reported a prevalence of 36% versus 71% in two species of rodents (*Jaculus jaculus* and *Jaculus orientalis*) in North Africa. It is clearly essential to screen more geographic regions for *Hepatozoon* prevalence, to improve estimates of diversity, to assess potential trophic pathways and to identify which genetic lineages can be found in diverse hosts. When this can be combined with microscopic assessments, there is an added benefit that morphological variability in gamonts can be assessed and parasitaemia levels recorded.

## Materials and methods

In this study, liver tissue samples (stored in 96% ethanol) from 43 Bushveld gerbils (*Gerbilliscus leucogaster*) from three neighbouring farms in the Free State province, South Africa (27°50’ S 26°03’ E), were screened for *Hepatozoon* using PCR amplification and sequencing of a partial 18S rRNA fragment typically used to detect and identify these parasites (O’Dwyer et al. 2013). Single samples from three potential predators that were found as road kills were also assessed: one adult female Cape fox (*Vulpes chama*) found on R34, close to Hoopstad, Free State province (27°43’11.8” S 25°48’50.4” E); one adult female small-spotted genet (*Genetta genetta*) found close to Molepo Dam, Limpopo province; and one adult male slender mongoose (*Galerella sanguinea*) found close to Blouberg Nature Reserve, Limpopo province. Molecular methodologies followed standard procedures (e.g. Harris, Maia & Pereira 2011), but in brief consisted of DNA extraction using a high-salt methodology, followed by PCR amplification using HepF300 and HepR900 (Ujvari, Madsen & Olsen 2004), with PCR cycling consisting of 94 °C for 30 s, 60 °C for 30 s and 72 °C for 1 min, with 35 cycles. Negative and positive controls (from Harris et al. 2011) were run for each reaction, and products were sequenced on both strands. Sequences were aligned against a data set of previously published *Hepatozoon* sequences (from Maia et al. 2014).

Maximum likelihood (ML) and Bayesian inferences (BI) were used to estimate phylogenetic relationships. ML analysis was performed using the software PhyML 3.0 (Guindon et al. 2010), with support estimated using the bootstrap technique with 1000 replicates. jModeltest 0.1.1 was used to choose the best model of evolution (Posada 2008). BI was implemented using MrBayes v3.1 (Huelsenbeck & Ronquist 2001), with the same model (GTR+I+G) and run for one million generations. After 25% burning, remaining trees were combined in a 50% majority rule consensus. *Haemogregarina balli* and *Dactylosoma ranarum* were defined as outgroups, following Maia et al. (2014).

For microscopy, 13 samples of gerbil blood smears were available. Slides were air-dried, fixed with pure methanol and stained with Giemsa following Telford (2009). Microscopy was conducted at 400X magnification using an Olympus CX41 microscope. Each slide was examined for at least 10 min, whereupon if no parasites were observed it was recorded as negative. Because in mammals *Hepatozoon* gametocytes occur almost always in leukocytes, primarily neutrophils, blood-smear examination was focused on the ‘feather edge’, where these cells are usually found.

## Results

**FIGURE 1:**
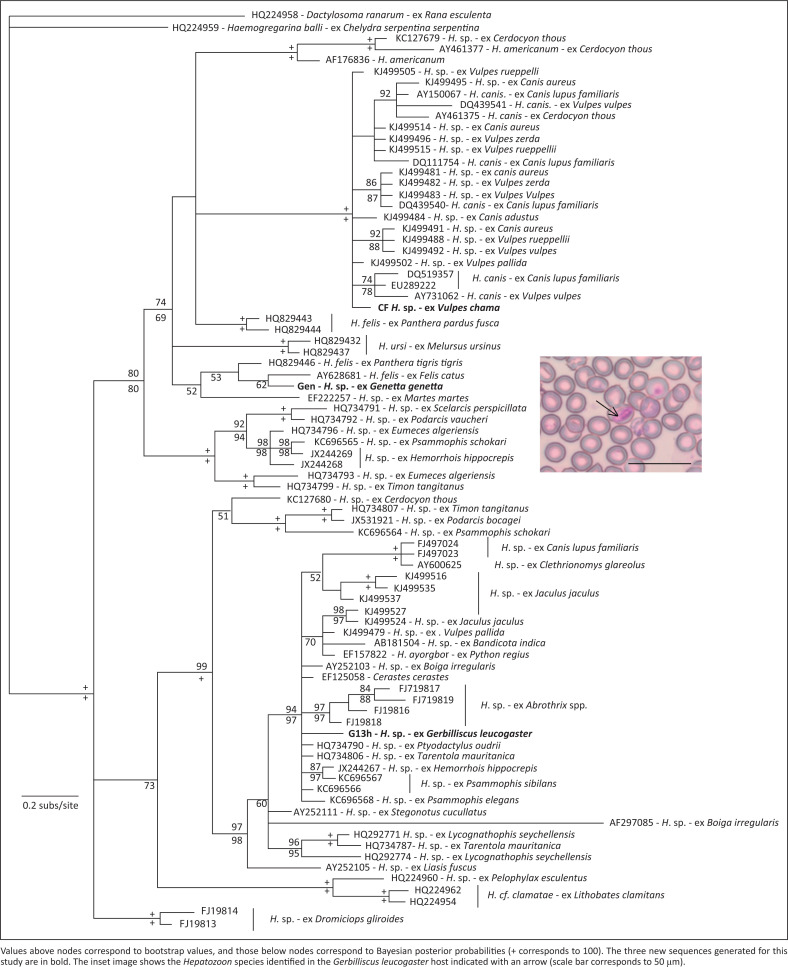
Estimate of relationships of *Hepatozoon* species derived from partial 18S rRNA using ML.

Based on the molecular screening, three samples were positive, and all were found to be infected with *Hepatozoon* (Figure 1). Overall, the estimate of relationships is similar to previously published phylogenies (e.g. Maia et al. 2014). The single Cape fox sample was infected, and the recovered sequence falls within a clade of sequences identified as *H. canis*. The most similar sequence is from an infected pale fox (*Vulpes pallida*) from Mauritania (one nucleotide difference). The single genet sample was also infected, and this sample is a sister taxon to a sequence from a parasite identified as *H. felis* from a domestic cat. Although various studies using microscopy have identified genets as a host for *Hepatozoon* (e.g. Averbeck et al. 1990; Keymer & Brocklesby 1971), this is to our knowledge the first confirmation using molecular techniques that the type of parasite is apparently *H. felis*. Again, this demonstrates that, at least with respect to the vertebrate host, there is very little host specificity, with *H. felis* (or *H. felis*-like) parasites not only common in felids, but here identified in a member of the family Viverridae. Only one of the gerbils was infected (sample code G13H), and this sample falls within a clade comprising samples from various small mammals, but also snakes (Figure 1). This is a surprisingly low infection rate compared with previous assessments of rodents (e.g. Bajer et al. 2014; Maia et al. 2014). Examining the blood slides, however, confirmed this – only a single slide, corresponding to the same individual, was positive. Furthermore, parasitaemia was very low, with only one in 10 000 counted cells infected (Figure 1).

## Discussion

These results allow us to draw several new inferences regarding *Hepatozoon* infections. Firstly, this is the first confirmation that genets can be infected with what is apparently *H. felis*, based on the molecular data. Secondly, we confirm the presence of *H. canis* in the Cape fox in South Africa, with a genetic lineage very similar to that found in foxes in North Africa. Finally, the rodents tested were mostly negative, unlike other studies that have found a high prevalence in rodents. The single positive sample, the first sequenced from this host species, showed a clear relationship with other *Hepatozoon* species from other rodent hosts. Thus, the major clades (Figure 1) are reinforced by additional sampling from this geographic region. Molecular assessments of *Hepatozoon* in tortoises from South Africa have recently revised taxonomic conclusion (e.g. Cook et al. 2014), and our results indicate that assessment of mammals, even when collected opportunistically for other studies, can give valuable new information concerning parasites that have both wildlife conservation and veterinary implications, although greater sampling would be valuable to extend this study.
